# Metal Sounds Stiffer than Drums for Ears, but Not Always for Hands: Low-Level Auditory Features Affect Multisensory Stiffness Perception More than High-Level Categorical Information

**DOI:** 10.1371/journal.pone.0167023

**Published:** 2016-11-30

**Authors:** Juan Liu, Hiroshi Ando

**Affiliations:** Center for Information and Neural Networks (CiNet), National Institute of Information and Communications Technology and Osaka University, Osaka, Japan; Kyoto University, JAPAN

## Abstract

Most real-world events stimulate multiple sensory modalities simultaneously. Usually, the stiffness of an object is perceived haptically. However, auditory signals also contain stiffness-related information, and people can form impressions of stiffness from the different impact sounds of metal, wood, or glass. To understand whether there is any interaction between auditory and haptic stiffness perception, and if so, whether the inferred material category is the most relevant auditory information, we conducted experiments using a force-feedback device and the modal synthesis method to present haptic stimuli and impact sound in accordance with participants’ actions, and to modulate low-level acoustic parameters, i.e., frequency and damping, without changing the inferred material categories of sound sources. We found that metal sounds consistently induced an impression of stiffer surfaces than did drum sounds in the audio-only condition, but participants haptically perceived surfaces with modulated metal sounds as significantly softer than the same surfaces with modulated drum sounds, which directly opposes the impression induced by these sounds alone. This result indicates that, although the inferred material category is strongly associated with audio-only stiffness perception, low-level acoustic parameters, especially damping, are more tightly integrated with haptic signals than the material category is. Frequency played an important role in both audio-only and audio-haptic conditions. Our study provides evidence that auditory information influences stiffness perception differently in unisensory and multisensory tasks. Furthermore, the data demonstrated that sounds with higher frequency and/or shorter decay time tended to be judged as stiffer, and contact sounds of stiff objects had no effect on the haptic perception of soft surfaces. We argue that the intrinsic physical relationship between object stiffness and acoustic parameters may be applied as prior knowledge to achieve robust estimation of stiffness in multisensory perception.

## Introduction

Humans extract environmental properties from multiple sources of sensory information. Haptic sensations are usually accompanied by contact sounds. From the “ding” of a metal plate, the “clunk” of a wood table, or the “bong” of a drum, we can often tell what the object is made of, its size, how it is being struck, and even imagine the tactile sensation that occurs when striking it [1, 2]. If we tap a surface and obtain sound feedback that differs from our expectation, will our haptic sensation be altered along with the sound? How is contact sound integrated with haptic signals to form a coherent percept of the object? Do the object properties inferred from sounds (e.g., the material from which the object is made) play a major role, or are the acoustic parameters of the sounds themselves more influential?

Although extensive studies have been performed to examine human abilities of deriving object attributes from impact sounds, such as inferring material composition [3–6], hollowness [7], shape [8], size [9] and the hardness of the hammer or the sounding-object [1, 2], there are relatively few studies on the interaction of auditory and somatosensory processing. Some audio-haptic experiments in which participants were asked to match the haptic property of roughness and the audio properties of loudness have shown that participants tend to associate louder sounds with rougher textures and softer sounds with smoother textures [10]. Guest et al. [11] found that high-frequency amplification led to increased perception of roughness and dryness. Touch and auditory cues about surface texture were weighted 62% and 38% respectively in a bimodal roughness judgment task [12], and their weights were task-dependent [13, 14]. However, the audio inputs used in roughness experiments have been mostly band-limited noise or sounds of rubbing sandpaper, which do not include object information such as material composition, object shape, or size. Audio-haptic interaction with abundant information on sound sources inferred from acoustic signals was not explored in these studies.

In the field of virtual reality, researchers have noticed the influence of sound on the haptic stiffness sensation [15–17]. The rankings of the stiffness of simulated haptic surfaces when presented along with recorded or synthesized sounds increased with the auditory stiffness. Recent work has shown that when no tactile cues were available, the audio feedback of a tapping action made participants perceive that the tapped virtual surface was softer for weak sounds [18]. Auditory information might also affect the perception of body parts [19]. Hitting participants’ hands gently and providing a temporally correlated sound of a hammer hitting a piece of marble could induce the feeling of a stiffer and heavier hand [20].

However, the interaction between auditory and somatosensory information is not yet fully understood. On the basis of anatomical, electrophysiological and neuropsychological evidence, it is reasonable to assume that the neural networks for auditory perception are organized hierarchically from spectrotemporal feature detection to more general object categorization [21]. In both monkey and human experiments, neural correlates of categorical perception of sound stimuli have been found [22]; that is, large areas of auditory cortex are sensitive to low-level acoustic features, but some areas can encode the categorical information that remains invariant even when the acoustic features vary. We classify such categorical information as high-level properties of sound in the sense that they emerge from the combination of low-level features and might have specific neural representations. Our question is: if an interaction exists between auditory and somatosensory information in stiffness perception, do auditory properties at different levels interact equally with somatosensory information? More specifically, in this study we investigated how the perceived information of contact sounds influences humans’ impressions of stiffness in haptic tasks, with special attention to the effects of high-level knowledge of material category and the low-level acoustic properties of frequency and damping parameters.

We conducted three experiments to estimate the influence of different auditory features on the haptic perception of surface stiffness. We first assessed whether the perceived stiffness would change along with the contact sounds of different material categories in audio-haptic tasks, where haptic stimuli at different stiffness levels were provided. Next, we adjusted the frequency and damping parameters while maintaining the category property in Experiments 2a and 2b, based on previous work in sound source perception [23, 24], to test whether high-level material knowledge or low-level acoustic features were more influential in multisensory stiffness perception. We also compared the results with those of audio-only conditions to examine whether they showed the same tendency. To present various combinations of haptic information and contact sounds that can induce the realistic feeling of tapping an object, we used a force-feedback device and employed the modal synthesis method [25, 26] to generate the impact sounds coupled with the timing and strength of participants’ tapping actions. Instead of playing back recorded sounds, we extracted acoustic parameters from the sampled impact sounds of real objects so that we could independently manipulate the parameters of acoustic models to synthesize the desired impact sounds and examine the effects of different levels of auditory information. This study will highlight how the influence of auditory properties at different levels on stiffness perception varies in uni- and multi-sensory conditions.

## Experiment 1: Effect of contact sounds of objects made from different materials

### Material and Methods

In this experiment, participants were asked to remember and reproduce the stiffness of standard surfaces with different contact sounds in an adjustment task.

#### Participants

Eight right-handed participants (four females, mean age ± standard deviation (SD): 38 ± 6 years) took part in Experiment 1. All participants had normal or corrected to normal vision, normal hearing, and reported no known motor deficits. The experiment was conducted according to the principles in the Declaration of Helsinki, and was approved by the ethics committee of the National Institute of Information and Communications Technology. All participants provided their written informed consent prior to the experiment.

#### Stimuli and apparatus

**Fig 1:**
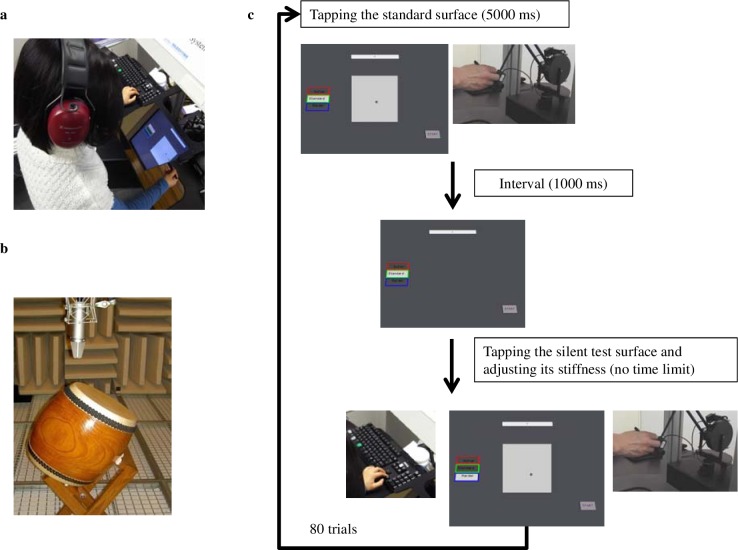
Experimental apparatus and procedure. (a) The experimental setup consisted of a CRT display showing visual stimuli, which were viewed through a mirror, and a force-feedback device, which was concealed beneath the mirror with its reference point calibrated at the center of the reflected display surface. (b) The contact sound recorded in an anechoic chamber was used to extract a set of modal frequencies for synthesizing the impact sound in correspondence with participants’ tapping actions. (c) The procedure of Experiment 1. When participants held the stylus and struck the standard surfaces (a light gray square), they could hear different contact sounds via headphones. Their left hands manipulated the keyboard to adjust the stiffness of a silent test surface while their right hands were holding the stylus and tapping the surface.

The haptic stimuli were presented with a force-feedback device, PHANToM Premium 1.5A (Geomagic, formerly Sensable, Rock Hill, SC, USA), and were developed using Reachin API (Reachin Technologies, Hässelby, Sweden) which ran on a Dell Precision 690 computer with a 3.0-GHz Xeon processor and a Creative Sound Blaster X-FiTM audio card. The virtual surfaces that participants tapped with the stylus of the PHANToM were light gray squares that were used only to show the tapping area and did not change their appearance during the experiment. Participants wearing shutter glasses could see the virtual surfaces and a dark gray sphere representing the movement of the stylus through a mirror reflecting the three dimensional (3D) images rendered on a 17-inch CRT monitor (refresh frequency 100 Hz, resolution 1024 × 768 pixels) as shown in Fig 1A. In our experiments, the stiffness of the virtual surfaces was adjusted by varying a parameter in the Reachin API from 1 N/m to 1000 N/m. Therefore, participants experienced different force feedback when tapping the different virtual surfaces. The contact force was computed at the rate of a haptic rendering routine (1 kHz), using a force model generated at the 100 Hz scene graph loop rate. Meanwhile, the force signals were fed into the audio excitation model to drive sound models at a rate of 44.1 kHz. The delay between sound and force was in the min–max range [0.833, 4.300] ms (mean ± SD: 0.984 ± 0.585 ms), which was much shorter than the Just Noticeable Difference (JND) of 24 ms for temporal haptic-audio asynchrony [27]. Participants wearing Sennheiser HDA 200 headphones could hear the contact sounds and feel the touch of the surfaces without perceivable delay.

The sound model in the physically motivated modal synthesis method is **M** = {**f**, **d**, **a**}, which describes a vibrating object by a bank of damped harmonic oscillators with modal frequencies *f*_*n*_, damping parameters *d*_*n*_, and amplitudes *a*_*n*_, where the mode number *n* = 1, …, *N* [28]. The vibrating object is taken as a linear time-invariant system (LTI), which can be characterized by its impulse response *h(t)*.

$$h(t)=\sum_{n=1}^{N}a_{n}e^{-d_{n}t}\sin(2\pi f_{n}t)$$ (1)

Contact sound *y(t)* is generated as the convolution of the impulse response *h(t)* and the input force *x(t)*, i.e.,

y(t)=(h*x)(t) (2)

Impact sounds for sound model measurement were recorded in an anechoic room (Fig 1B) so that environmental sound effects such as room acoustics were not contained in the samples. We recorded the impact sounds of three objects: tapping the head of a Japanese drum with a wooden stick, tapping the wooden body of the drum with a wooden stick, and tapping a copper alloy plate with a plastic pen. The head of the Japanese drum was made of leather (cowhide). Hereafter, these sounds are referred to as *drum*, *wood* and *metal*. The parameters of modal frequencies were estimated by fitting them to the sampled sound file using the method described in our previous work [26]. For each sound model, 15 modal frequencies were selected from the parameter regression results. Although spatialized sounds have been reported as an important manipulation in multisensory studies because of the importance of spatial congruency in multisensory integration [29–31], because sound source localization was not the main issue of this study, we synthesized and reproduced monaural sounds in all of our experiments.

#### Stiffness adjustment task

Participants were seated in front of the force-feedback device and looked into the mirror that reflected the monitor image. As shown in Fig 1C, they were asked to tap a standard surface for 5 seconds and remember its stiffness. Then, the standard surface disappeared and after a 1-second blank screen, a silent test surface was shown. The participants’ task was to adjust the stiffness parameter with the keyboard until they felt that the stiffness of the test surface was the same as that of the previous standard surface. There were two kinds of stiffness levels (hard surface: 700 N/m; soft surface: 200 N/m) and four kinds of sound conditions (no contact sound, metal, wood, drum), for a total of eight kinds of standard surfaces. The initial stiffness of the silent test surfaces was a random value in the range [1, 1000] N/m. Participants could choose a large (± 50 N/m) or small (± 1 N/m) step size when adjusting the stiffness parameter. There was no time limit for the adjusting process, and the next trial began when participants pushed a button to confirm the result of the present trial. Experiment 1 consisted of 10 trials for each of the eight experimental conditions presented in pseudo-randomized order, for a total of 80 trials. For each participant, the mean value of the 10 trials of each condition was taken as a sample for the statistical analysis. All of the participants completed the experiment in 1 hour. Two-way repeated measures analysis of variance (RM-ANOVA) were performed with surface stiffness and sound as factors.

After finishing the task, we assessed informally the ability of participants to identify the material of the sound-generating objects. All of the participants could differentiate among the contact sounds and infer the three kinds of gross material categories: metal, wood, and drum (stretched leather) that could make the sounds, which were the same as the materials of the objects that we used to sample the sounds.

### Results

**Fig 2:**
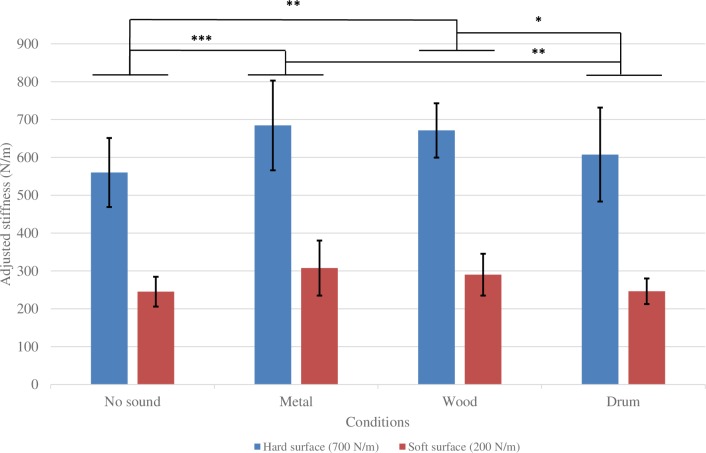
Results of Experiment 1: Adjusted stiffness of each condition. **Error bars show 95% confidence intervals adjusted by the Bonferroni method.** *: .01 < p < .05, **: .001 < p < .01, ***: p < .001.

Fig 2 shows the mean adjusted stiffness of the participants, along with 95% confidence intervals (CI). Participants reproduced significantly different surface stiffness in the hard surface and the soft surface conditions (F_1,7_ = 98.643, p < .0001). Meanwhile, the sound condition turned out to be significant (Greenhouse-Geisser corrected value: F_1.719, 12.035_ = 8.018, p = .008). However, no interaction effect existed between the surface stiffness and contact sound conditions (Greenhouse-Geisser corrected value: F_1.585, 11.094_ = 0.68, p = .494). Multiple comparisons of the four sound conditions using the Ryan’s procedure showed that metal and wood sounds enhanced the feeling of a hard surface compared with the no-sound condition. No-sound–Metal = –93.376 N/m, p < .001; No-sound–Wood = −78.095 N/m, p = .002. The drum sound condition did not differ significantly from the no-sound condition (p = .284), but differed significantly from the metal (p = .005) and wood (p = .023) sound conditions.

Experiment 1 indicated that the contact sounds of different material categories had different effects on haptic stiffness perception. Surfaces with wood and metal sounds were perceived as harder than no-sound surfaces, while surfaces with the drum sound were not significantly different from no-sound surfaces. Nevertheless, it is too early to conclude that the material category is the main factor in auditory information that influenced haptic stiffness perception. First, the categories in Experiment 1 were very different from one another, but at the same time the acoustic parameters (e.g., frequency and damping) of different materials were also distinct from each other. It is not clear which factor actually influenced the results of stiffness adjustment, the material category or the lower-level acoustic features. Second, because the participants adjusted the stiffness of a test surface after the exposure to the standard stimulus had ceased, the adjustment task was strongly memory-dependent, which would have reinforced the role of categorical processing. Furthermore, the participants could not precisely reproduce the stiffness of standard surfaces even in the no-sound condition. Our *t*-test results showed that the participants appeared to under-estimate the hard surface (T_7_ = –3.623, p = .008) and over-estimate the soft one (T_7_ = 2.726, p = .03) in the no-sound condition, which could be a tendency toward the mean stiffness level when their judgments were made under uncertainty. Therefore, we conducted Experiments 2a and 2b to address these issues.

## Experiments 2a and 2b: Material category vs. acoustic parameters

The first modification from Experiment 1 was that lower-level acoustic parameters were modulated to examine which factor, material category or acoustic parameters, was more relevant to the audio-haptic integration process. The second modification was that we used two approaches to reduce the memory-dependency of the adjustment task in Experiment 1. Experiment 2a still applied the stiffness adjustment task, but the standard surface and the test surface were presented side by side so that participants could tap both of them during the adjustment procedure. Experiment 2b was a stiffness comparison task, in which the participants made their judgment immediately after their exposure to the two stimuli and did not have to remember the haptic feeling of the standard surfaces for a long time. We conducted both of them to test whether different experimental procedures would influence the results. In each experiment, the audio-only tasks were also carried out to examine whether auditory information has the same effects in unisensory and multisensory tasks of stiffness perception.

### Material and Methods

#### Participants

There were 10 participants (four females, mean age ± SD: 28.3 ± 5.1 years) in Experiment 2a. Another nine participants (three females, mean age ± SD: 33.2 ± 8.8 years) were recruited for Experiment 2b. All participants were right-handed, and had normal or corrected to normal vision, normal hearing, and reported no known motor deficits. They were paid for their participation and were naïve to the purpose of the experiment. All experiments were conducted in accordance with the recommendations of the ethics committee of the National Institute of Information and Communications Technology with written informed consent from all participants. All 19 participants provided their written informed consent in accordance with the Declaration of Helsinki prior to the experiments.

#### Sound stimuli

We chose metal and drum sounds whose mode frequencies were wide apart for Experiments 2a and 2b. The mode parameters of the two original sounds (M1, D1), which were the metal (M) and drum (D) sounds used for Experiment 1, are listed in the table in S1 Table, available online. In Wildes & Richards’ study [23], a coefficient of internal friction tan φ (= *d /* (*πf*), *d*: damping, *f*: frequency) was proposed to account for the identification of gross material categories. If we modified (M1, D1) to (M2, D2) by adjusting the centroids of their mode frequencies *f*_*c*_ (Eq 3) to be the same, i.e., *f*_*c*_^*M2*^ *= f*_*c*_^*D2*^, while maintaining the internal friction coefficients (tan φ) constant, then the modified sounds (M2, D2) as shown in Eqs 4 and 5 could be perceived as being from the same categories of material as those of the original sounds.

fc=∑nanfn∑nan (3)

$$\alpha f_{c}^{M1}=f_{c}^{M2}=f_{c}^{D2}=\beta f_{c}^{D1}$$ (4)

$$d_{n}^{M2}=\alpha d_{n}^{M1},\;d_{n}^{D2}=\beta d_{n}^{D1}$$ (5)

In our experiments, *f*_*c*_^*M1*^ = 2430.73 Hz, *f*_*c*_^*D1*^ = 283.56 Hz. We chose the coefficients as *α* = 0.21, *β* = 1.80.

**Fig 3:**
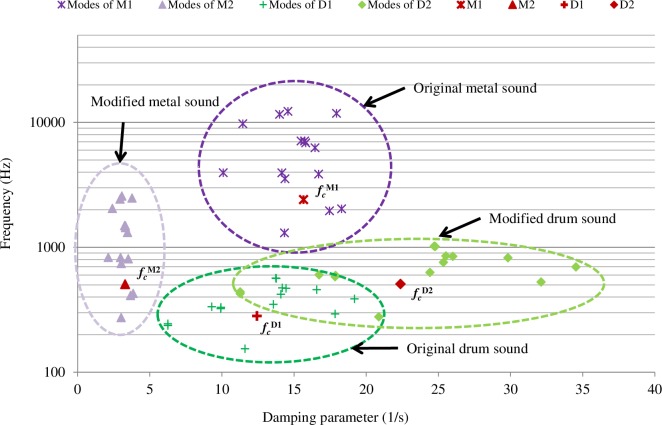
Modes of the original (M1, D1) and modified (M2, D2) sounds.

The distribution of modes of the original and modified sounds in the frequency-damping space is shown in Fig 3. Experiments using these original and modified sounds would make it clearer which factors of auditory information were integrated with haptic inputs in the stiffness perception. To verify the assumption that the material categories of modified sounds were perceived the same as the original sounds, all of the participants of Experiments 2a and 2b completed a material categorization task.

#### Material categorization task

This was an audio-only task where the experimenter tapped virtual surfaces to produce different impact sounds, and participants wearing headphones chose the material category of the sounding-source and reported their confidence rating (*CR*) from 1 (low) to 7 (high) points. The categories to choose from were drum (A), metal (B), or others (C). When participants chose category C, they could write down the material such as glass, wood, plastic, and so on if they had one in mind. There were 50 types of sound, half of which were modified from the original metal sound M1 and the other half from the original drum sound D1. We manipulated the mode frequency *f*_*n*_ and damping parameters *d*_*n*_ of sound models *i* and *j* as shown in Eqs 6 and 7.

$$f_{n}^{i}=l_{i}f_{n}^{M1},\;d_{n}^{i}=k_{i}d_{n}^{M1},\;i=1,\ldots ,25$$ (6)

$$f_{n}^{j}=l_{j}f_{n}^{D1},\;d_{n}^{j}=k_{j}d_{n}^{D1},\;j=1,\ldots ,25$$ (7)

Therein, the elements of coefficient set {*l*_*i*,_ *k*_*i*_} were values from {0.1, 0.21, 0.5, 1.0, 1.2}, and the elements of coefficient set {*l*_*j*_, *k*_*j*_} were values from {0.6, 1.0, 1.4, 1.8, 2.2}. So, when {*l*_*i*,_ *k*_*i*_} was {1.0, 1.0}, the sound was the original metal sound M1. When {*l*_*i*,_ *k*_*i*_} was {0.21, 0.21}, the corresponding sound was sound M2 used in Experiments 2a and 2b. When {*l*_*j*_, *k*_*j*_} was {1.0, 1.0}, the sound was the original drum sound D1 and {1.8, 1.8} produced the sound D2 used in Experiments 2a and 2b. The score of each sound was the sum of the confidence rating of each category (*CR*_*A*_, *CR*_*B*_, *CR*_*C*_) across all participants. There was one trial for each of the 50 sounds, which was presented in pseudo-randomized order, and no time limit was set for response.

#### Experiment 2a: Audio-haptic and audio-only stiffness adjustment task

Experiment 2a was composed of two stiffness adjustment tasks with a similar experimental setting to that in Experiment 1. In the audio-haptic adjustment task, the standard surface and the test surface were presented side by side, and the participants could tap them back and forth during the adjustment until they thought that the test surface was adjusted as hard as the standard surface. In the audio-only task, the experimenter would tap the standard surface to produce a synthesized impact sound, and the participants wearing headphones would imagine the stiffness from the auditory information, tap the test surface and adjust it to the imagined stiffness level. Participants could ask the experimenter to generate the sound again during the adjustment. The sound conditions were determined by a factorial combination of two factors–material category and acoustic parameters (four kinds of sound: M1, M2, D1 and D2). By combining these sound conditions with two different surface stiffness levels (Hard, Soft), we produced eight kinds of standard surfaces (HM1, HM2, HD1, HD2, SM1, SM2, SD1, SD2). Typical examples of the impact sounds that resulted from tapping these surfaces are available in the Supporting Information online. This task consisted of eight trials for each of the eight experimental conditions presented in pseudo-randomized order. The audio-haptic session took 28.14 min on average, with standard deviation 10.33 min, while the audio-only session took 25.00 min on average, with standard deviation 8.50 min. For each participant, the mean value of the eight trials of each condition was taken as a sample for statistical analysis. Three-way repeated measures analyses of variance (RM-ANOVA) were performed with surface stiffness, material category, and acoustic parameter as factors.

#### Experiment 2b: Audio-haptic and audio-only stiffness comparison tasks

In Experiment 2b, we applied Scheffé’s paired comparison method [32] for both the audio-haptic and the audio-only tasks. This method was developed to obtain a ranking of stimuli at an interval scale. Based on the variances and degrees of freedom in the data, a ‘yardstick’ is produced to determine a minimum distance in the ranking that denotes a statistically significant difference between conditions based on a chosen significance level (5% in this experiment). Because the two standard stiffness levels in Experiment 1 could be distinguished easily, we increased the number of stiffness levels of the surfaces to three: 700 N/m, 450 N/m, and 200 N/m, to make the task reasonably challenging. By combining the three levels of stiffness factor with the two sound factors (four kinds of sound: M1, M2, D1 and D2), we obtained 12 experimental conditions in total. In the audio-haptic stiffness comparison task, we asked the participants to tap two surfaces, one after the other, and judge the difference in perceived stiffness between the two surfaces on a five-point ordinal scale according to the haptic feeling. Surfaces were shown for 3 seconds with a 500-ms interval between Surface 1 and Surface 2. The five possible response options were:

Surface 1 is much harder than Surface 2: 2
Surface 1 is slightly harder than Surface 2: 1
Surface 1 is as hard as Surface 2: 0
Surface 2 is slightly harder than Surface 1: −1
Surface 2 is much harder than Surface 1: −2

Each of the possible pairs of the 12 experimental conditions (including order of presentation) was presented once, in random order, for a total of 132 trials. The two values of the same pair with a different order of presentation were averaged, and the results were analyzed using the Nakaya variation of Scheffé’s paired comparison [33]. The audio-only stiffness comparison task followed the same procedure, except that the experimenter tapped the two surfaces while the participants wearing headphones listened to the impact sound and made the five-point response verbally.

#### Hypothesis

In Experiment 1, three factors could account for the result that the surfaces with sound M1 were perceived as stiffer than the surfaces with sound D1: (1) M1 is a metal sound, whereas D1 is a drum sound; (2) M1’s frequency is higher than that of D1; (3) M1’s damping parameter is larger than that of D1. According to the relationship among the acoustic parameters in the four sounds, for Experiments 2a and 2b we could make three different predictions under the assumption that the material category, frequency or damping parameters was the main factor, respectively: The order of surface stiffness should be M1 = M2 > D2 = D1 if the material category dominated the multisensory integration; however, if a lower-level feature were more important, because the spectral centroid of M2 and D2 were almost the same, the order would be M1 > M2 = D2 > D1 when frequency played the main role, and D2 > M1 > D1 > M2 when damping parameter was the key factor. It was also possible that Experiments 2a and 2b would yield different results if the audio-haptic integration strategy changed across tasks.

### Results

**Fig 4:**
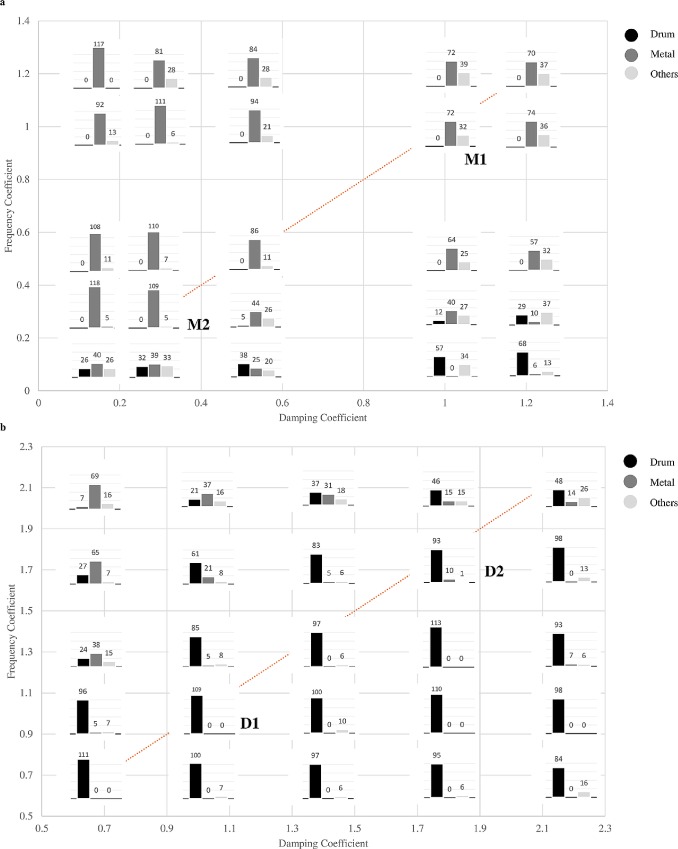
Results of the material categorization task. (a) Material categorization of the sounds modified from the original metal sound (M1) using frequency coefficient *l*_*i*_ and damping coefficient *k*_*i*_. (b) Material categorization of the sounds modified from the original drum sound (D1) using frequency coefficient *l*_*j*_ and damping coefficient *k*_*j*_. The three numbers (*CR*_*A*_, *CR*_*B*_, *CR*_*C*_) above each sub-figure indicate the score of the sound, i.e., the sum of the confidence rating of each category (Drum, Metal, Others) across all participants. Because only one category could be chosen for each trial, the maximal score of *CR*_*A*_ + *CR*_*B*_ + *CR*_*C*_ of the 19 participants was 19 × 7 = 133. Sounds along the dotted line were the sounds in which tan φ was kept the same as that of the original sound.

Fig 4 presents the results of the material categorization task where the frequency coefficients and damping coefficients in Fig 4A and 4B were {*l*_*i*,_ *k*_*i*_} and {*l*_*j*,_ *k*_*j*_} in Eqs (6) and (7), respectively. The data indicated that the material category could be maintained by modulating frequency and damping parameters simultaneously in a certain range. The score of sound M1 was (0, 72, 32), and that of sound M2 was (0, 109, 5). The *Metal* category was chosen with high confidence level, and no participant chose the *Drum* category for these two sounds. When we only reduced the frequency to that of M2 but kept the damping unchanged, i.e., the coefficient set was {0.21, 1.0}, half of the score was not in the *Metal* category. Interestingly, the value of the *Others* category for sound M1 was larger than that of M2, and the participants who reported this category all wrote “glass”. Also, the other three sounds near M1 in Fig 4A were categorized in the same way. The result was consistent with the work of Giordano and McAdams [6], where steel was perceptually equivalent to glass, and these two were taken as one gross category. In Fig 4B the score of sound D1 was (109, 0, 0) and that of sound D2 was (93, 10, 1). Nearly 90% of the points were in the *Drum* category. Again, when we only increased the frequency but kept the damping unchanged, i.e., the coefficient set was {1.8, 1.0}, the sound was not categorized as *Drum* any more for most participants. These results supported our assumption for Experiments 2a and 2b using sounds M1, M2, D1, and D2 that the material category of a sound could be kept intact if the frequency and damping parameters were modulated carefully. It is noteworthy that keeping tan φ constant to maintain the material category of the sounding sources did not work with all coefficient sets. For sounds modified from the metal sound M1, when the coefficient of frequency was 0.1, the sound was too low to be recognized as a metal sound. Also, for sounds modified from the drum sound D1, when the coefficient of frequency was 2.0, the sound source was very rarely inferred to be a drum.

**Fig 5:**
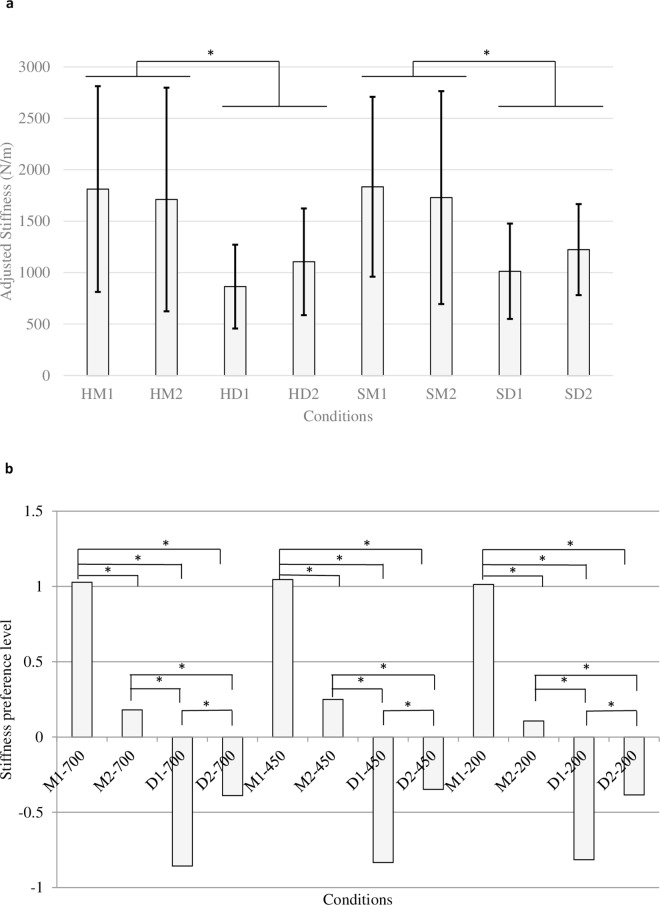
Results of audio-only tasks in Experiments 2a and 2b. (a) Results of audio-only adjustment task. Error bars show 95% confidence intervals adjusted by the Bonferroni method. (b) Results of audio-only Scheffé’s paired comparison with yardstick Y_.05_ = 0.1585. *: .01 < p < .05.

The results of the audio-only conditions of Experiments 2a and 2b shown in Fig 5 demonstrated similar characteristics of sound-induced stiffness perception. In the stiffness adjustment task (Fig 5A), the main effect of material category was significant (F_1,9_ = 8.228, p = .0185), which shows that the metal sounds always gave an impression of a stiffer surface than the drum sounds in the audio-only condition. There was no main effect of surface stiffness (F_1,9_ = 4.890, p = .0543) and acoustic parameter (F_1,9_ = 0.747, p = .410). Participants did not imagine the stiffness of sounds made by tapping hard surfaces differently from that of sounds made by tapping soft surfaces. In the paired comparison task (Fig 5B), sounds made by virtual surfaces of different stiffness levels (700 N/m, 450 N/m, 200 N/m) did not produce significantly different preference levels. However, there were significant differences between each pair of the four kinds of sound (F_11, 440_ = 364.302). The yardstick Y_.05_ was 0.1585, which means that if the difference of preference values exceeds 0.1585, the two ratings differ significantly (p < .05). The preference order of stiffness was M1 > M2 > D2 > D1. Again, in this audio-only task, both the original and the modified metal sounds were perceived as stiffer than the drum sounds.

**Fig 6:**
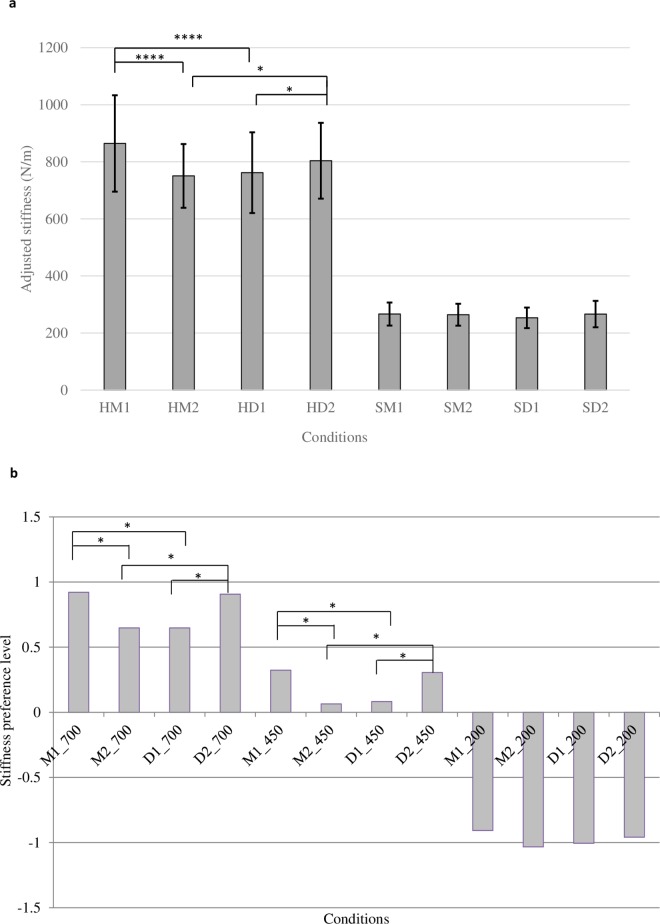
Results of audio-haptic tasks in Experiments 2a and 2b. (a) Results of audio-haptic stiffness adjustment task. Error bars show 95% confidence intervals adjusted by the Bonferroni method. (b) Results of audio-haptic Scheffé’s paired comparison with yardstick Y_.05_ = 0.1674. *: .01 < p < .05, ****: p < .0001.

It seemed reasonable to assume that the above effect of sound on stiffness perception in audio-only conditions would also appear in audio-haptic tasks together with a significant effect of the stiffness level of surfaces. Indeed, the effect of the stiffness level was supported by our data. In the audio-haptic stiffness adjustment task (Experiment 2a), the adjusted stiffness of the test surfaces was significantly different for the two kinds of stiffness level (700 N/m and 200 N/m), despite the presence of contact sounds (Fig 6A), F_1, 9_ = 75.959, p < .0001. In the paired comparison task, surfaces of different stiffness levels (700 N/m, 450 N/m, 200 N/m) were significantly different in their preference levels, shown in Fig 6B (F_11,440_ = 368.65). The yardstick Y_.05_ was 0.1674.

To our surprise, we found that the effects of sound were not the same in the audio-haptic and audio-only tasks. In the audio-haptic adjustment task, the main effects of material category (F_1,9_ = 1.175, p = .223) and acoustic parameter (F_1, 9_ = 1.902, p = .201) were not statistically significant, but there were interaction effects between the material and acoustic parameter factors, F_1,9_ = 23.810, p = .0009, and among the stiffness, material and acoustic parameter factors, F_1,9_ = 26.268, p = 0.0006. Post-hoc analysis using Ryan’s procedure showed that in the hard surface condition, surfaces with the original metal sound M1 were perceived as much stiffer than those with the original drum sound D1 (F_1,36_ = 24.761, p < .0001), which was consistent with the results of Experiment 1 and the audio-only conditions. Further, the surfaces with M1 elicited a significantly stiffer impression than did those with the modified metal sound M2 (F_1,36_ = 36.967, p < .0001). Meanwhile, the surfaces with the modified drum sound D2 also produced a significantly stiffer impression than did those with the original drum sound D1 (F_1,36_ = 5.015, p = .0314). However, comparing the data of M2 with those of D2, we found that surfaces with the modified metal sound M2 elicited a significantly softer impression than did those with the modified drum sound D2 (F_1,36_ = 6.635, p = 0.0143). This was opposite to the results of the audio-only adjustment task. None of these effects in the hard surface conditions were found in the corresponding soft surface conditions.

The results of the paired comparison task in Experiment 2b showed that in the 700 N/m and 450 N/m conditions, the stiffness preference of surfaces with D2 was significantly higher than that of those with D1, and even reached the level where no significant difference was found from that of M1, which suggested a stronger effect of acoustic parameters than that in the audio-haptic adjustment task. Conversely, the stiffness preference of surfaces with M2 was significantly lower than that of those with M1, and had no significant difference from that in those with D1. The order of stiffness preference turned out to be M1 = D2 > M2 = D1, in which the positions of D2 and M2 were reversed compared with those in the audio-only condition. As in the audio-haptic adjustment task, no such effects of sound were found in the 200 N/m conditions.

**Fig 7:**
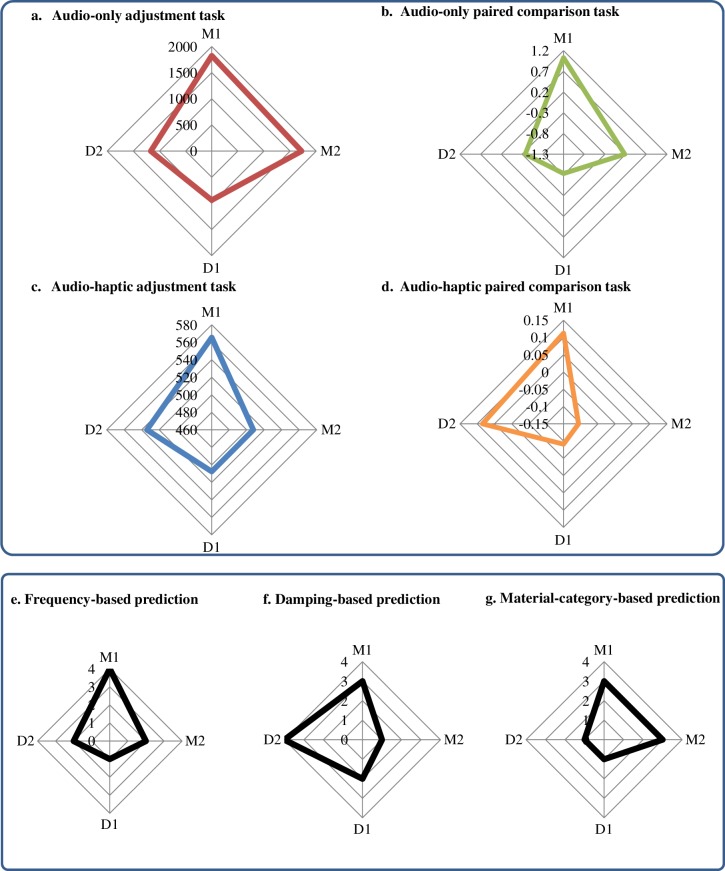
Stiffness of the four sounds (M1, D1, M2, D2). Results of (a) audio-only adjustment task with values of adjusted stiffness (N/m); (b) audio-only stiffness comparison task with values showing the stiffness preference level; (c) audio-haptic stiffness adjustment experiment with values of adjusted stiffness (N/m); and (d) audio-haptic stiffness comparison task with values of stiffness preference level. These values are the averaged data across all of the haptic stiffness levels in each experiment. (e)–(g) show the predictions of the order of stiffness according to frequency (e), damping (f) parameters and material category (g). The values from 0 to 4 represent the order of stiffness from soft to hard.

To compare the results with our predictions of the stiffness order based on three auditory features (material category, frequency and damping parameters), we averaged our data across all of the haptic stiffness levels for the four sounds (M1, M2, D1, D2) in each experiment. As can be seen in Fig 7, when participants only had the auditory information, they estimated (Fig 7A) or judged (Fig 7B) the stiffness by combining material-category (Fig 7G) and frequency (Fig 7E) features. When the auditory information was integrated with the haptic information in audio-haptic tasks (Fig 7C and 7D), both frequency and damping parameters became influential. In particular, the relationship between D2 and M2 resembled that of the damping-based prediction (Fig 7F), and was opposite to the material-category-based prediction (Fig 7G).

Consistent with this observation, the Pearson product-moment correlation coefficients between the experimental results and each feature-based prediction (see Table 1) indicated that frequency-based prediction was positively associated with the results of all four tasks, whether audio-only or audio-haptic. However, the material-category-based prediction was strongly related to the audio-only tasks and weakly related to the audio-haptic tasks. In contrast, the damping-based prediction was related strongly to the results of the audio-haptic tasks and weakly to those of the audio-only tasks.

**Table 1:** Pearson product-moment correlation coefficient *r* of the experimentally obtained results and the predictions based on three auditory features.

	Auditory feature		
	Frequency (Fig 7E)	Damping (Fig 7F)	Material category (Fig 7G)
Exp. 2a audio-only stiffness adjustment (Fig 7A)	0.802	-0.236	0.971
Exp. 2b audio-only paired comparison (Fig 7B)	0.956	0.033	0.870
Exp. 2a audio-haptic stiffness adjustment (Fig 7C)	0.911	0.643	0.330
Exp. 2b audio-haptic paired comparison (Fig 7D)	0.730	0.875	0.031

## Discussion

The present results demonstrated that haptic stiffness perception was susceptible to auditory information, but the effect of contact sounds in the audio-haptic condition was not the same as that in the audio-only condition. These findings suggest that auditory features are used differently in unisensory and multisensory processes for stiffness judgment. We found two characteristics of audio-haptic stiffness perception, which are distinct from that of audio-only stiffness perception.

First, the magnitude of the auditory effect on multisensory stiffness perception varied with the haptic stiffness level. In the audio-haptic tasks of Experiments 2a and 2b, the auditory effect was not significant when the sounds of stiff objects were presented with soft surfaces (200 N/m). One may suppose that sound amplitude accounts for this, because the synthesized sound was calculated from the convolution of contact force and sound models. A softer surface would generate a weaker force than would a hard surface, so that the amplitude of its contact sound was lower, and in turn the effect of sound would be less obvious. However, the data of the audio-only condition showed that the sounds made by soft surfaces (200 N/m) were not perceived significantly differently from those made by hard surfaces (700 N/m). Furthermore, in the medium stiffness condition (450 N/m) of Experiment 2b, the auditory effect was as strong as that in the high stiffness condition (700 N/m). This indicates that the variation of the auditory effect was not consistent with the change in sound amplitude, and the two are probably not causally related. A plausible explanation might be that the force feedback of soft surfaces (200 N/m) and the contact sound of stiff objects were not taken as signals generated by the same event, so the discrepant multisensory signals were not integrated effectively. As suggested in the studies of depth cue combination [34] and visuo-haptic integration [35], the nervous system may discount conflicting information if the discrepancy between individual estimates is outside the limits typical of the physical world.

Second, not all of the auditory features could be tightly integrated into the haptic processing flow. In our audio-only tasks, either the original or the modified metal sounds were judged to be stiffer than the original or modified drum sounds. Because stiffness is closely related to the material composition of an object, material category might be the most reliable property extracted from the auditory signal when participants are asked to estimate or judge stiffness from sound. This is in line with observations by Giordano et al. [1], i.e., that the most accurate specifier of sounding-object hardness is tan φ, which indicates the material-related information. However, in the audio-haptic condition, we could modulate the acoustic parameters to make participants feel that a touched surface with a drum sound was stiffer than the same surface with a metal sound. A similar effect was observed in both the multisensory adjustment (Experiment 2a) and pairwise comparison (Experiment 2b) tasks, which shows that this audio-haptic interaction for stiffness perception was stable across tasks. The influence of auditory material-related information on haptic perception has been studied in the domain of virtual reality and ecological perception (see a thorough review by Giordano and Avanzini [36]). The ratings for the stiffness of audio-haptic events were usually found to increase with the auditory stiffness of real [15] and synthesized [16] sounds. By adjusting low-level acoustic parameters while maintaining the material-related information, we found that the auditory effect was not always consistent in the audio-only and audio-haptic conditions.

The difference between the unisensory and multisensory results suggests that the low-level acoustic features, other than the material category, were deeply involved in the haptic processing flow, and the audio-haptic interaction might originate in a mandatory perceptual process [37], because the biases occurring in multisensory tasks were against participants’ subjective impressions of the sound alone. The interaction of low-level acoustic features and haptic information may happen at an early stage in the processing flow; there is increasing neuroscientific evidence pointing to multisensory integration at very early sensory processing levels [38]. It has been reported that the rat posterior insular cortex contains an interposed and overlapping region capable of integrating auditory and somatosensory signals [39]. The posterior and lateral side of the primary auditory cortex of the macaque monkey has been implicated as the site for the integration of auditory broadband noise and tactile stimulation of the hand and foot [40]. A human fMRI study [41] has also shown that auditory and somatosensory inputs converge in a sub-region of human auditory cortex along the superior temporal gyrus when a somatosensory stimulus is passively provided and no haptic tasks are assigned. Audio-haptic stiffness judgment tasks in our experiments may involve the neural substrates of somatosensory perception, such as the parietal operculum [42] and the region near the supra-marginal gyrus [43], which are activated during hardness-related haptic tasks.

We also note that in the same material category, sounds with higher frequency and/or shorter decay time (large damping parameter) gave an impression of stiffer objects, which was common in the audio-only and audio-haptic conditions. The concordance among participants in associating the somatosensory perception of stiffness with acoustic features seems to reflect the regularities of the physical environment. In our tasks, the quantity defining stiffness was the restoring force that participants could feel from the stylus when tapping a virtual surface. Physically, the restoring force is mainly determined by material stiffness and tension, which are also two important factors for vibration and impact sounds. The stiffness or tension of a plate and a membrane is in direct proportion to the square of the object’s vibration frequency [44], i.e., stiffer objects often generate impact sounds with higher frequencies. Consistently with Freed’s observation [2] that the spectral centroid mean was the strongest predictor of the perceived mallet hardness, our results showed that the order of the spectral centroids of the four sounds (M1, M2, D1, and D2) was positively correlated with the judgment of stiffness in both unisensory and multisensory tasks. However, the damping parameter is closely related to material composition [2, 5, 6, 24] as well as to the impact event. Chaigne and Doutaut’s work [45] shows that the contact time of impact decreases with an increase in the stiffness of either the hammer or the sounding object, and it has been reported that a contact-time-related parameter in an audio model of impact influences auditory and haptic perception of stiffness [16]. A large damping parameter results in a rapid decay and short duration of the contact sound, which is likely to be associated with the haptic feeling of a short impact that usually happens when tapping a stiff object. The intrinsic relationship between low-level acoustic features and physical stiffness might be acquired from concurrent somatosensory and auditory information, and used as prior knowledge for stiffness perception to achieve robust estimation of object properties.

Although stiffness and acoustic parameters are closely related, object structure also causes a change in acoustic parameters, e.g., a longer bar produces impact sounds with lower frequency. Therefore, the estimation of object stiffness cannot be uniquely determined by acoustic parameters. Since humans can infer structural properties from contact sounds, such as size [9, 46, 47], 3-D shape [48], and hollowness [7], it is possible that such structural information is taken into account in audio-haptic stiffness judgments as well. These effects need to be further investigated.

## Supporting Information

S1 File Example of the original metal sound (M1) generated by tapping the hard surface (700 N/m). (WAV)

S2 File Example of the modulated metal sound (M2) generated by tapping the hard surface (700 N/m). (WAV)

S3 File Example of the original drum sound (D1) generated by tapping the hard surface (700 N/m). (WAV)

S4 File Example of the modulated drum sound (D2) generated by tapping the hard surface (700 N/m). (WAV)

S5 File Example of the original metal sound (M1) generated by tapping the soft surface (200 N/m). (WAV)

S6 File Example of the modulated metal sound (M2) generated by tapping the soft surface (200 N/m). (WAV)

S7 File Example of the original drum sound (D1) generated by tapping the soft surface (200 N/m). (WAV)

S8 File Example of the modulated drum sound (D2) generated by tapping the soft surface (200 N/m). (WAV)

S9 File Data of Experiment 1. (XLSX)

S10 File Data of Experiment 2a. (XLSX)

S11 File Data of Experiment 2b. (XLSX)

S12 File Data of material categorization task. (XLSX)

S1 Table Modes of the original metal sound (M1) and the original drum sound (D1) in Experiments 2a and 2b. (DOC)
