# Supplementary material for: Metal Sounds Stiffer than Drums for Ears, but Not Always for Hands: Low-Level Auditory Features Affect Multisensory Stiffness Perception More than High-Level Categorical Information
Source: PLoS One. 2016 Nov 30;11(11):e0167023. doi: 10.1371/journal.pone.0167023 (PMC5130246; doi:10.1371/journal.pone.0167023)
Supplement: S1 Table — (DOC) [file pone.0167023.s013.doc]

**Metal Sounds Stiffer than Drums for Ears, but Not Always for Hands: Low-level Auditory Features Affect Multisensory Stiffness Perception More Than High-level Categorical Information**

Juan Liu* & Hiroshi Ando

Center for Information and Neural Networks (CiNet), National Institute of Information and Communications Technology and Osaka University, Osaka, Japan.

* Corresponding author

E-mail: juanliu@nict.go.jp (JL)

**S1 Table. Modes of the original metal sound (M1) and the original drum sound (D1) in Experiments 2a and 2b.**

The sound model in the physically motivated modal synthesis method is $\mathbf{M}=\{\mathbf{f,d,a}\}$, which describes a vibrating object by a bank of damped harmonic oscillators with modal frequencies $f_{n}$, damping parameters $d_{n}$ and amplitudes $a_{n}$, where the mode number $n=1,...,N$. The vibrating object is taken as a linear time-invariant system (LTI), which can be characterized by its impulse response:

$h(t)=\sum_{n=1}^{N} a_{n}e^{-d_{n}t}\sin(2\pi f_{n}t)$.

Contact sound $y(t)$ is generated as the convolution of the impulse response and the input force $x(t)$, i.e.,

$y(t)=(h*x)(t)$.

In our experiments, 15 modes were used for each sound.

| **Mode number** | **Metal** | | | **Drum** | | |
| --- | --- | --- | --- | --- | --- | --- |
|  | ***f(Hz)*** | ***d(1/s)*** | ***a*** | ***f(Hz)*** | ***d(1/s)*** | ***a*** |
| 1 | 1308.14 | 14.33 | 1.00 | 154.50 | 11.59 | 1.00 |
| 2 | 1964.90 | 17.45 | 0.70 | 237.40 | 6.26 | 0.55 |
| 3 | 2040.27 | 18.29 | 0.24 | 388.14 | 19.18 | 0.53 |
| 4 | 3967.49 | 10.08 | 0.11 | 350.45 | 13.56 | 0.43 |
| 5 | 3865.21 | 16.69 | 0.09 | 244.94 | 6.25 | 0.22 |
| 6 | 11848.65 | 17.94 | 0.05 | 293.93 | 17.84 | 0.19 |
| 7 | 3956.73 | 14.15 | 0.03 | 474.81 | 14.18 | 0.12 |
| 8 | 7122.11 | 15.73 | 0.03 | 459.73 | 16.56 | 0.09 |
| 9 | 3547.60 | 14.37 | 0.03 | 471.04 | 14.44 | 0.08 |
| 10 | 6282.31 | 16.45 | 0.02 | 331.61 | 9.93 | 0.05 |
| 11 | 7127.49 | 15.48 | 0.02 | 324.07 | 9.91 | 0.04 |
| 12 | 9786.84 | 11.44 | 0.02 | 335.38 | 9.29 | 0.04 |
| 13 | 12295.46 | 14.57 | 0.01 | 422.05 | 14.08 | 0.04 |
| 14 | 11644.08 | 13.99 | 0.01 | 565.25 | 13.76 | 0.02 |
| 15 | 6874.48 | 15.80 | 0.01 | 569.01 | 13.73 | 0.02 |
